# Rural migration, governance, and public health nexus: Implications for economic development

**DOI:** 10.3389/fpubh.2022.1002216

**Published:** 2022-10-11

**Authors:** Kewen Yang, Shah Fahad

**Affiliations:** ^1^College of Economics and Management, Northwest A&F University, Xianyang, China; ^2^School of Management, Hainan University, Haikou, China

**Keywords:** rural migrant workers, parental health, governance, mediation analysis, economic development

## Abstract

With the deepening of rural aging and the increasing role of human capital in the non-agricultural employment labor market, this paper uses the data of China Health and Retirement Longitudinal Study (CHARLS), ordinary least squares (OLS) and instrumental variable method (IV) to try to examine the impact of rural migrant workers' education on their parents' health. Since a rural family may include more than one child, a sample of migrant workers with a high education level is used in the benchmark regression, and a sample of migrant workers with a low education level is used to test the robustness of the relationship. The results showed that the education of migrant workers had a significant positive impact on parents' health. The sample with the least education was used for the robustness, and the results did not change. The IV-probit method is used to address potential endogeneity, and the results remain stable. Heterogeneity analysis shows that there are significant differences in the impact of migrant workers' education on the health of parents from different groups. This positive effect has a greater impact on the health of parents who are older, less educated, and do not live with their children. Mediation analysis shows that children's economic ability, captured by income and work type, and their parents' health behavior, captured by sleep, alcohol consumption, and physical examinations, mediate this relationship. Thus, migrant workers' education affects their parents' health mainly through relaxing budget constraints and improving their parents' health production efficiency. In addition, this paper also found that education of migrant workers may significantly increase parental depression. Based on the above analysis, this paper argues that increasing investment in rural education is conducive to improving the health of migrant farmers' parents, thereby promoting the transfer of rural labor to non-agricultural industries and cities, curbing the rapid rise in labor costs, and promoting the healthy development of the economy.

## Introduction

According to the standards of the United Nations, China has been an aging society since 1999, and the degree of aging is increasing. In 2016, the number of older people (65 years and above) in China was 150 million, representing 10.9% of the total population. In 2019, the 65- and older population reached 176 million, representing 12.6% of the total population (data from the National Bureau of Statistics). At the same time, due to the long-term flow of rural young and middle-aged labor to urban and non-agricultural industries, the aging degree of rural areas is higher, and the pension problems faced by the elderly in young and middle-aged outflow families are more severe.

Elderly people in China have long been supported mainly by their families (1). Traditionally, China follows the “back-feeding model”, with adult children supporting their parents (2). In recent years, with the gradual establishment and improvement of China's social security system, an increasing number of elderly people place low economic demands on their children and some elderly people even provide their children with financial support (3). However, in rural areas, due to limited social security, the elderly rely mainly on their children for economic support. According to census data for 2010, 66.3% of urban elderly people rely on pensions as their main source of income, while 47.7% of rural elderly people rely mainly on other family members for support (2).

With China's social and economic development, more and more rural young people migrate to cities and non-agricultural industries, making their parents into pension difficulties. According to the data of migrant workers monitoring survey report, in 2016, the total number of migrant workers in China reached 280 million people, of which about 170 million people left their hometowns, and about 80 million migrant workers moved across provinces, accounting for 45.3% of the total number of migrant workers leaving their hometowns. The massive transfer of these rural labor forces, who bear the main responsibility of support, on the one hand, reduces the daily care for their parents; on the other hand, parents have to do more farm work, housework, and need to help migrant workers take care of their children, which reduces their health and satisfaction of life (4). Farmers' going out to work has impacted the foundation of family pension, making their parents face pension difficulties.

The intergenerational support children provide their parents includes economic resource support, daily care, and emotional comfort (2). Although daily care and emotional comfort have an important impact on elderly parents, the support of economic resources is more important for rural parents (5). According to the 2014 China Longitudinal Aging Social Survey, the poverty rate of expenditure in rural areas reached 38.0%. Although children who migrate to work provide less daily care and emotional comfort for their parents, they can obtain more economic income through work, and thus provide more financial resources for their parents. Li's (6) research shows that 70.3% of migrant workers send money home, and the average amount of money sent or returned is 2,576 yuan. These remittances are sustainable and play an important role in supporting rural families. The proportion of peasant households whose remittances represent more than 50% of total household income has reached 46.3%. On average, the amount of remittances is ~40% of the total household income. To some extent, economic resource support not only compensates for lack of daily care and emotional comfort, but is also conducive to the development and improvement of the social care system (2).

According to human capital theory, people with higher education levels are more competitive in the labor market than those with lower education levels (7). The flow of rural laborers into cities and non-agricultural industries is affected by restrictions on the household registration system and other adverse conditions, which lead to an increase in migration costs (8). Additionally, in the labor market, the level of income and the quality of work are affected by the human capital stock (7). It is shown that, as an important part of human capital, a higher level of education leads to greater knowledge reserves, greater labor skills, a greater ability to overcome the mobility restrictions of the household registration system, a lower cost of migration, and a higher likelihood of finding higher income and better work, all else held constant (8). Therefore, this paper will examine the pension problems of migrant workers' parents from the perspective of education.

Health problems are one of the main problems faced by the elderly. After entering old age, people will show deterioration of health status, loss of labor ability and daily living ability at the physiological level, and easily fall into psychological difficulties such as frustration, depression, anxiety, and fear at the psychological level (9). When children migrate to work, their parents' health problems become more prominent (2). Therefore, if the health of the elderly can be improved and the morbidity rate can be reduced, it will not only reduce the pain caused by the disease, improve the happiness and quality of life of the elderly, but also help maintain the harmony and stability of the family. In view of this, this paper will focus on parents' pension in relation to health issues. In theory, the higher the education level of migrant workers, the richer the economic resources and health knowledge, the more conducive to the improvement of parents' health from the economic level and health behavior level.

Previous studies have examined the relationship between child education and their parents' health (10–15). For example, De Neve and Harling (16) used data from South Africa to examine the relationship between rural children's education and their parents' mortality risk. They found that children's education reduced the mortality of their parents. However, their study was mainly a correlation analysis and did not identify a causal effect between children's education and their parents' health. To identify such an effect, Ma (17) used data from the China Health and Retirement Longitudinal Study (CHARLS) of 2011 and instrumental variables constructed based on the 9-year mandatory education policy to investigate the causal effect of children's education on their parents' health. The results showed that children's education significantly improved cognitive function, lung function, and survival expectations of their parents. According to the analysis of possible influencing mechanisms, this positive effect can be attributed to the fact that a higher education level helps children provide financial support for their parents, improve their resource acquisition ability, reduce their labor supply, and improve their psychological welfare. Yang et al. (9) used the same data and reached the same research conclusion using different instrumental variables.

However, the above research still has three shortcomings. First, existing studies have mainly examined the general population, and no studies have examined migrant workers. As we all know, since the reform and opening, migrant workers have made important contributions to the rapid development of China's economy. In the future, with the decline of the total population, especially the working-age population, migrant workers will still play an important role in the high-quality development of China's economy. However, whether farmers can successfully realize the transfer from rural and agricultural to urban and non-agricultural industries is affected by the health of their parents (18). Therefore, special research is necessary on this group. Second, there is little literature on the heterogeneous impact of children's education on the health of different groups of parents. It is easy to ignore the dynamic response of parents to their children's influence, which is not conducive to a comprehensive grasp of the relationship between children's education and parents' health. Third, only a small number of literature have examined the specific impact mechanism, but these literatures focus mainly on the economic resource level and pay insufficient attention to health behavior. Due to the high correlation between economic resources and health behavior, ignoring the impact of health behavior is easy to overestimate the role of economic resources and ignore the benefits of health behavior.

Therefore, this paper will seek to answer the following three questions: first, does the education of migrant workers affect the health of their parents? Second, is there heterogeneity in this effect among groups of parents with different characteristics? Third, what are the specific influencing mechanisms of this effect? The main contributions of this paper to the existing literature are as follows. First, there are few studies on the causal effect between children's education and the health of their parents. Using the instrumental variable method, this paper studies migrant workers from rural areas, which is helpful in testing the universality of children's education on parents' health effects. Furthermore, in the context of the socioeconomic development and transformation of China, the study of the impact of migrant workers' education on their parents' health can provide a solid empirical basis for the transfer of the rural labor force. Second, from the perspective of children's economic situation and parental health behaviors, this article examines the channels through which the education of migrant workers can affect the health of their parents. On the one hand, the study compensates for the shortcomings of existing research; on the other hand, it provides a practical basis for guiding children's education to improve their parents' health. Third, with the deepening of aging in China, the study of parental health is conducive to the realization of healthy aging, which is of great practical significance for the construction of a harmonious society.

The rest of this paper is arranged as follows: The second part outlines the theoretical hypothesis, the third part describes the research design and introduces the data used, the fourth and fifth parts concern the empirical analysis, and the last part presents the research conclusion, discussion, and policy recommendations.

## Theoretical hypothesis

Although the traditional concept of pensions is changing with the development of industrialization and urbanization, in many societies, the family is still the means of support for the elderly (1). Especially in East Asia, there is a strong tradition of filial piety. Parents and children support each other, and young people respect the elderly. Furthermore, in China, these traditional values are consolidated by the government in the form of legislation that stipulates that adult children have the responsibility to take care of their parents (19). This “filial piety culture” based on the double constraints of morality and the legal system enables parents to obtain unconditional support from their children, which is reflected in the flow of resources from children to their parents.

Economic support is the most important type of intergenerational support that migrant children provide to their parents. This support includes economic resources, daily care, and emotional comfort (2). In rural areas, due to limited social security and limited savings, children provide the main source of income for their older parents (2). For migrant children, economic support is important because economic resources substitute for daily care and emotional comfort. Because their migrant work physically separates them from their parents, migrant children offer reduced daily care and emotional comfort to their parents, and economic support is the most important means of intergenerational support (5). Du et al. (20) showed that compared to children who do not migrate to work, children who migrate to work provide more financial support to their parents. However, this does not mean that migrant children no longer provide any daily care or emotional comfort to their parents. With the development of modern information technology and new means of transportation, migrant children can contact and communicate more easily with their parents, which to some extent reduces the negative impact of inadequate emotional comfort caused by the separation of living spaces (21).

The concept of family support for the elderly is deeply rooted in China. With the deepening of aging, intergenerational support has become an important factor affecting the health of parents when social care is insufficient (22). Empirical research shows that children's support of their parents has a significant effect on their parents' health regardless of whether objective health indicators, subjective health indicators, or cognitive indicators are used to measure parents' health (5, 20). For example, Zuo and Li (23) examined Chaohu data and found that the economic support of migrant children has an important impact on their parents' psychological wellbeing and self-assessed health and compensates for the negative impact of living space separation to some extent. Lian et al. (4) used data from the China Health and Nutrition Survey to study the relationship between child labor migration and their parents' health and life satisfaction and found that child labor migration led to a decrease in their parents' health and life satisfaction. This finding was attributed to the impact of child labor migration on the traditional family pension model and the reduced daily care and comfort children offer their parents along with the increase in the time parents must engage in agriculture and family care. However, this paper did not conduct an empirical study of the impact mechanism. Liu (21) research in rural areas in China showed that child labor migration does not automatically decrease the welfare of their parents. In fact, it is a mutually beneficial network of support among family members, rather than migrant work of children, that affects the welfare of parents. When there is no supportive network among family members, children's labor migration reduces intergenerational support for parents, but as long as such a network exists, children's labor migration can still have a positive impact on the welfare of their parents.

According to the theory of intergenerational exchange, the child's support of their parents is influenced by the support of their parents' resources (9), including not only short-term resource exchange, such as daily care provision in exchange for financial support (24), but also long-term support. In a long-term resource exchange, the investment of parents in their children's early education is an important means of intergenerational support that has a long-term effect on the intelligence, knowledge and skills of children. Having a higher education level offers children a higher chance of obtaining a good job and high income in the labor market when they are adults (7) and the ability to obtain health knowledge and maintain a healthy lifestyle. Therefore, in return for the investment of their parents, educated children may be more willing and able to support their parents in old age (25, 26). In contrast, parents' underinvestment in their children's education and the resulting low level of education in the early stage of life greatly increase the likelihood of children becoming “NEET” in adulthood (27). On the basis of the above analysis, this paper proposes the following theoretical hypothesis.

*Hypothesis 1: With other influencing factors unchanged, the higher the education level of migrant workers, the better the health of their parents*

The influence of the education of migrant workers on their parents' health is affected not only by the skills of migrant workers, but also by the abilities of their parents. The education and age of parents are important factors that affect their responses to external influences. From the perspective of children, whether they live with their parents is an important factor that affects their ability to influence the health status of their parents. Therefore, it is necessary to focus on the heterogeneity of the impact of migrant worker education on the health of their parents according to education level, living arrangements, and age.

Regarding education, Zimmer et al. (10) believe that parents with higher education can use more effectively the resources of migrant workers (such as income and health knowledge) to improve their own health, so the education of migrant workers has a greater impact on the health of parents with higher education. However, more educated parents may have more resources on their own and be less likely to need resources from their children. In contrast, parents with low educational levels are more in need of support from migrant workers due to their poor ability to obtain resources and their limited ability to improve their health; consequently, the education of migrant workers may have a greater impact on the health of parents with lower educational levels. Lee and Xiao (24) showed that parents with lower income and status have poorer health and receive more support from their children. For the rural elderly in China, economic resources are important and scarce, so the impacts on the health of their parents with lower education are great.

From the perspective of age, the older the parents, the worse their health tends to be (28), and the more assistance they need from their children to complete physically demanding tasks. In addition, as their age increases, the declining income of parents and the smaller fluctuations in consumption can lead to greater material demands on their children. Family support is still the main means of support for the elderly due to incomplete institutional and market-oriented pension methods. Therefore, with the gradual increase in the age of parents, their ability to work and manage daily activities gradually decreases and the support that migrant workers provide to their parents increases (1).

Previous studies have shown that the labor market to which migrant workers have access is a typical dual employment market, and there is usually a division of employment and income (29). Generally, because migrant workers do not have non-agricultural household registration and their education level is low, they have poor employment prospects in the non-agricultural employment market and obtain low income (30). To support their families, they must, therefore, economize on food, housing, transportation, and other expenses, which leads to poor living conditions. When parents and their migrating children live together, the poor living environment may offset the health improvement provided by the income of the children. When parents do not live with their migrating children, they can not only enjoy a better living environment in their hometown, but also receive income from their children to improve their living standards and thus improve their health. Based on the above analysis, this paper proposes the following theoretical hypothesis:

*Hypothesis 2: The education of migrants has a greater impact on the health of parents with lower education levels and older ages. The ability of migrant workers to support the health of their parents when they live with their parents is limited*

The more educated migrant workers are, the more likely they are to obtain good jobs and income, and thus to be able to offer financial support to their parents (17). This support can provide their parents with not only a more balanced and reasonable diet but also improved physical and mental health through special health investments such as travel, vacations, and fitness opportunities (13).

Furthermore, having a high level of education usually means that an individual has strong learning ability and rich knowledge, including the mastery and identification of health-related knowledge and behaviors (13). Therefore, higher education migrant workers can share their own knowledge of health and have a positive external impact on their parents, thus improving the efficiency of allocation and utilization of their parents' health resources (12). From the perspective of allocation efficiency, the positive externality of migrant workers' education can help parents optimize their health investment and improve their health. From the perspective of efficiency of utilization, this means that the same input of health resources can lead to higher health outcomes, such as a better understanding of treatment plans, better coordination of treatment, and better treatment effects. Based on the above analysis, this paper proposes the following theoretical hypothesis.

*Hypothesis 3: Economic support and improvement in parental health behavior are important ways in which the education of migrant workers affects the health of their parents' health*

## Study design

### Model and methods

According to the data types used below, in order to reduce measurement errors, this paper converts the five-category dependent variables into two-category variables. To this end, this paper sets the following binary selection model (Probit) as a benchmark model to test the relationship between the education of migrant workers and the health of their parents.


(1) $$\begin{array}{l} Pr\left(Y_{i}=i\right)=\phi \left(\alpha +\beta E_{i}+\gamma X_{i}+\mu_{k}+u_{i}\right) \end{array}$$

where *i* represents an individual, *k* represents a province, *Y*_*i*_ is the health of parents, *E*_*i*_ represents the level of education of a migrant worker, *X*_*i*_ stands for other control variables, μ_*k*_ is the fixed regional effect, *u*_*i*_ is a term of random disturbance, β and γ are the coefficients of the corresponding variables.

### Data and variables

The research object of this paper is parents. In order to make the sample nationally representative, this paper uses the China Health and Retirement Longitudinal Study (CHARLS) data for research. This are because the data is currently the only nationally representative survey database for groups aged 45 and older in China, which is very suitable for this study. CHARLS was conducted by the China Center for Economic Research, National Institute of Development, Peking University, among randomly selected family members aged 45 and above. The survey has been implemented since 2011 and covers 150 county-level units, 450 village-level units, and 17,000 people in ~10,000 households. The samples were followed every 2 years, and the second wave in 2013 included new respondents in addition to those in 2011, increasing the total number of respondents to ~18,000. These data offer rich individual and family information and meet our research needs.

When collating the data, we first deleted individuals who were younger than 45 years of age. Second, we eliminated people whose children were under 16 years old or still in school. Third, given that the object of this study was migrant workers, we deleted individuals with non-agricultural household registration and retained individuals with agricultural household registration. On this basis, we deleted individuals whose occupation was farming and retained individuals engaged in non-agricultural work. Finally, the extreme values and missing sample points were removed. After data processing according to the above standards, the sample size for a single year was too small to meet the research needs of this article. Because the most recent data for the wave in 2015 did not include the variables related to children's occupation, the research object of this paper, we used only samples from 2011 and 2013 for the research data.

Since there may be more than one child in a single rural family, the data of one father or mother may correspond to the data of more than one child. Children with higher education levels may have higher social and economic status and more comprehensive health knowledge, and thus may have a greater influence on the health of their parents than children with lower education levels. Therefore, considering the former may better identify the relationship between the education of migrant workers and the health of their parents. Therefore, we adopted samples of high-education migrant workers to test the relationship. Furthermore, taking advantage of the fact that a rural family can have more than one child, this article also investigates the relationship between the low educational attainment of migrant workers and the health of their parents to test the robustness of the relationship.

The dependent variable in this paper is the health of migrant workers' parents. Health indicators can be roughly divided into objective indicators and subjective indicators. Compared to objective health indicators, subjective indicators, although simple, have been shown to predict mortality and disability rates (31). That is, it refers not only to the absence of disease or physical strength but also to physical and mental health, as well as social wellbeing and status. Taking advantage of the CHARLS database with rich health indicators, this article uses three types of indicators, namely self-assessed health (SAH), cognition, and depression, as proxy variables to more fully measure the health status of parents of migrant farmers.

In the CHARLS data of 2011 and 2013, respondents were randomly divided into two groups to obtain accurate sample values. At the beginning and end of the health-related questionnaire, the following two types of answers were used to assess health status. The first was “excellent, very good, good, fair, and poor” [corresponding to SAH (1) in Table 1], and the second was “very good, good, fair, poor, and very poor” [corresponding to SAH (2) in Table 1]. To reduce measurement error, “excellent, very good and good” and “very good and good” were assigned a value of 1, and “fair and poor” and “fair, poor, and very poor” were assigned a value of 0.

**Table 1:** Basic information of the sample.

**Variables**	**Obs**.	**Mean**	**Standard deviation**	**Min**	**Max**
**Parents' health**					
SAH (1)	25,028	0.272	0.445	0	1
SAH (2)	29,168	0.232	0.422	0	1
SAM	26,878	0.185	0.388	0	1
Minus7	29,954	0.343	0.475	0	1
Depressed	29,954	0.058	0.233	0	1
**Parental characteristics**					
Age	20,588	59.65	9.618	45	103
Male (female as control group)	23,653	0.477	0.499	0	1
Non-agricultural hukou (agricultural hukou as a control group)	23,600	0.201	0.401	0	1
Married (not married as a control group)	23,631	0.869	0.337	0	1
Years of education	20,597	7.425	2.315	6	19
Medical insurance (no insurance as control group)	29,709	0.947	0.225	0	1
Refrigerator (no refrigerator as control group)	15,849	0.849	0.358	0	1
**Migrant workers' characteristics**					
Years of education	13,552	10.69	3.336	6	22
Male (female as control group)	6,317	0.486	0.500	0	1
Living with parents (not living with parents as a control group)	6,132	0.149	0.356	0	1
Year	3,4043	2012	1.000	2011	2013
**Community characteristics**					
Socioeconomic status	33,857	3.792	1.349	1	7

The proxy variables for cognitive ability include self-assessed memory (SAM) and the correct number of consecutive subtractions of 7 (Minus7). To measure SAM, respondents were asked to subjectively rate their own memory as excellent, very good, good, fair, or poor. The answers of “excellent, very good and good” were assigned a value of 1 and the answers of “fair and poor” a value of 0. For the second proxy variable, the respondents were asked to calculate 100 minus 7, 93 minus 7, 86 minus 7, and so on for five consecutive subtractions. When the respondents gave the correct answer to all calculations, they received a value of 1; otherwise, 0.

Depression (depressed) was measured by whether the respondents had experienced the following feelings or behaviors in the previous week: “worrying about small things,” “difficulty concentrating when doing things,” “feeling depressed,” “difficulty doing anything,” “being full of hope for the future,” “being fearful,” “sleeping poorly,” “feeling happy,” “feeling lonely,” and “feeling unable to continue to live.” The possible answers were “little or no,” “not much,” “sometimes or half the time,” and “most of the time.” Among them, “feeling full of hope for the future” and “feeling happy” were reverse-coded. The responses of “most of the time” for these two indicators and “little or no” for the negative indicators yielded an overall value of 1; otherwise, 0.

The main explanatory variable of concern in this article is the education of rural migrant workers, which is represented by ordinal numbers 1–11. The value 1 indicates illiteracy, 2 indicates uncompleted primary school but the ability to read and write, 3 represents the completion of private school, 4 represents the completion of primary school, 5 represents the completion of junior high school, 6 represents the completion of senior high school, 7 represents the completion of vocational high school, 8 indicates college graduation, 9 represents a bachelor's degree, 10 represents a master's degree and 11 represents a doctorate degree. In this paper, values 1–4 are referred to as primary school and below. Because primary school graduates represent the largest proportion of respondents, the value is converted to represent 6 years of education; junior high school represents 9 years; senior high school and vocational high school represent 12 years; college graduates have 15 years; a bachelor's degree represents 16 years; a master's degree represents 19 years; and a doctorate degree represents 19 years of education. The same applies to parental education.

Other control variables included characteristics of the parents of the migrants, such as age, gender, type of household registration, marital status, education, medical insurance, and family economic status. The characteristics of the migrant workers included gender and whether they lived with their parents. In this paper, the socioeconomic status of the community in 2011 was used to measure the influence of community characteristics on the health of parents. A province dummy variable was used to express the different traditional cultural concepts, resource endowments, and economic development levels specific to each province. Thus, controlling for the regional fixed effect allowed us to alleviate the impact of unobservable factors on the results. We also controlled for the data survey year to control the impact of the time characteristics of different survey years on the model results. In China, people may have agricultural hukou, non-agricultural hukou, unified hukou, or no hukou. A very small number of people have no household registration (hukou), few people have unified household registration, and many people have agricultural household registration. In this paper, non-agricultural household registration is assigned the value 1, and the others are assigned the value 0. To better measure the socioeconomic conditions of families and increase the sample size, this paper considers whether there is a refrigerator at home as the proxy variable of socioeconomic conditions to reduce the possible measurement error that could affect the model estimation results if we used household wealth, income, consumption, or other direct measurement indicators. Other variables can be obtained directly from the CHARLS database and are not described in detail here.

### Basic information of the sampled respondents

The basic information in Table 1 reflects the descriptive statistical results of the health status and characteristics of parents, the characteristics of migrant workers, and the characteristics of the community. In general, the health status of parents of rural migrant workers is not ideal. The mean value of the calculation variable (Minus7) reaches only the level of 0.34. The averages for the other indicators are also poor, especially the index measuring depression. However, the values of these health indicators are relatively consistent, indicating that the measure of parental health is relatively robust. Average years of schooling among migrant workers amounts to ~11 years, indicating that rural migrant workers on average have a high school education. For parents, the average is ~7 years, indicating that parents on average have a junior high school education level. In general, migrant workers are more educated than their parents, which provides favorable conditions for their education to affect their parents' health.

## Empirical results and analysis

### Benchmark regression

According to the theoretical hypothesis and formula (1), this paper uses the binary selection model (Probit) to perform a benchmark regression, where the coefficient is the marginal effect. The estimated results are shown in Table 2.

**Table 2:** Benchmark model results.

**Explanatory variables**	**(1)**	**(2)**	**(3)**	**(4)**	**(5)**
	**SAH (1)**	**SAH (2)**	**SAM**	**Minus7**	**Depressed**
Migrant workers' years of education	0.005*	0.005**	0.006***	0.008***	0.003**
	(0.003)	(0.002)	(0.002)	(0.003)	(0.001)
Parents' characteristics
Age	−0.001	−0.000	−0.000	−0.004***	−0.000
	(0.001)	(0.001)	(0.001)	(0.001)	(0.000)
Male	0.050***	0.058***	0.057***	0.178***	0.008
	(0.015)	(0.014)	(0.013)	(0.015)	(0.008)
Non-agricultural hukou	0.001	0.068**	−0.056*	0.021	−0.000
	(0.034)	(0.031)	(0.029)	(0.037)	(0.011)
Married	−0.015	−0.010	−0.045***	0.037*	0.003
	(0.020)	(0.019)	(0.016)	(0.021)	(0.011)
Medical insurance	0.002	−0.060*	−0.028	0.276***	0.016
	(0.038)	(0.034)	(0.030)	(0.050)	(0.020)
Years of education	0.008*	0.006	0.010***	0.038***	0.001
	(0.004)	(0.004)	(0.003)	(0.004)	(0.002)
Refrigerator	0.046***	0.005	−0.007	0.086***	0.012
	(0.018)	(0.017)	(0.015)	(0.019)	(0.010)
**Migrant worker characteristics**					
Male	−0.006	0.005	−0.002	0.014	−0.002
	(0.015)	(0.014)	(0.012)	(0.015)	(0.007)
Living with parent	0.011	0.003	0.003	0.003	−0.004
	(0.020)	(0.019)	(0.017)	(0.021)	(0.010)
Year	−0.028***	−0.028***	−0.031***	−0.003	−0.008*
	(0.010)	(0.009)	(0.007)	(0.009)	(0.004)
**Community characteristics**					
Social economic status	0.017***	0.007	0.006	0.008	0.001
	(0.006)	(0.005)	(0.005)	(0.006)	(0.003)
Constant	193.653***	202.474***	281.540***	15.288	174.457*
	(68.585)	(62.617)	(68.131)	(59.075)	(91.613)
Province FE	Y	Y	Y	Y	Y
Observations	3,415	3,631	3,532	3,681	3,516
Wald statistical value	168.27	176.98	168.12	512.02	60.84
Pseudo-R^2^	0.048	0.049	0.059	0.118	0.042

Robust standard errors are in parentheses, ***P < 0.01, **P < 0.05, *P < 0.1.

The table shows that for each year increase in the length of schooling of migrant workers, the self-assessed health of their parents (SAH) (1) improves by 0.5%, SAH (2) improves by 0.5%, SAM improves by 0.6%, the precision of the calculation (Minus7) increases by 0.8%, and depression decreases by 0.3%. These results show that migrant workers' education has a significant positive impact on their parents' health.

The results for the control variables are basically in line with theoretical expectations. Regarding the characteristics of the parents of migrant workers, in general, the older the parents, the worse their health is. This is in line with the theory of health capital investment (28). Fathers tend to have better health than mothers. Household registration has different influences on different health indicators for the parents of migrant workers. The SAH (2) of parents with non-agricultural household registration is better than that of parents with other household registration types, but differences in household registration types do not have a significant influence on SAH (1). It also has a different influence on cognitive ability. The SAM of those with non-agricultural household registration is poor, but there is no significant effect on the accuracy of the calculation (Minus7). In general, it is difficult to determine the effect of the type of household registration on the health of parents of parents of migrant workers' parents. Marital status has different effects on different indicators of cognitive ability between parents. Parents who are married indicate a poor SAM but have a higher calculation accuracy (Minus7). Medical insurance also has different influences on different health and cognitive indicators. Parents with medical insurance have a poorer SAH (2), but there is no significant effect on SAH (1). In general, the impact of medical insurance on the health of parents of migrant workers is difficult to determine, possibly due to the relatively crude data available on medical insurance. The more years of education parents of migrant workers have completed, the better their health. Household economic conditions, measured by whether the family has a refrigerator, have a significant effect on the health of the parents of migrant workers. From the perspective of the characteristics of migrant workers, there is no difference in the impact of the gender of children on the health of their parents. Whether children live with their parents does not affect the health of the parents. Over time, the health of the parents of migrant workers worsens, which is consistent with the theory of capital investment. From the perspective of community characteristics, the higher the socioeconomic status of the community, the better the health of parents. This may be because a community with a high socioeconomic status has rich medical resources and health facilities and a green and sanitary environment, which are factors that promote good health.

### Robustness test

In the benchmark regression, the sample of migrant workers with the highest education level was used to investigate the relationship between the education of migrant workers and the health of their parents. The results showed that the education of migrant workers can significantly improve the health of their parents. If the impact of the education of migrant workers on their parents' health is stable, the relationship should also be supported using the sample of migrant workers with a lower level of education. Therefore, the sample of migrant workers with a lower education level is adopted to test the robustness of the studied relationship (the coefficient is the marginal effect). The results are shown in Table 3.

**Table 3:** Robustness test results.

**Explanatory variables**	**(1)**	**(2)**	**(3)**	**(4)**	**(5)**
	**SAH (1)**	**SAH (2)**	**SAM**	**Minus7**	**Depressed**
Migrant workers' years of education	0.010***	0.004	0.003	0.012***	0.004**
	(0.003)	(0.003)	(0.003)	(0.004)	(0.002)
Control variables	Control	Control	Control	Control	Control
Province FE	Y	Y	Y	Y	Y
Observations	3,537	3,748	3,652	3,801	3,729
Wald statistical value	162.29	156.60	118.42	522.43	67.29
Pseudo-R^2^	0.043	0.041	0.040	0.112	0.046

Robust standard errors are in parentheses, ***P < 0.01, **P < 0.05, *P < 0.1, respectively. The control variables (as shown in Table 2) and the constant terms are not listed.

The table shows that for every year increase in the level of education of migrant workers, the SAH (1) of their parents improves by 1.0%, the SAH (2) improves by 0.4%, the SAM improves by 0.3%, the accuracy of the calculation (Minus7) improves by 1.2% and depression decreases by 0.4%. Among them, only the coefficients for SAH (1), calculation accuracy (Minus7), and depression are significant, while those for SAH (2) and SAM are not significant. However, the results obtained using the sample of migrant workers with a low education level still show that the education of migrant workers has a significant effect on the health of their parents. The results for the control variables are basically in line with the theoretical expectations and differ little from the benchmark results presented above, so they are not described in detail here.

### 2SLS method

In the previous analysis, we used samples of migrant workers with different levels of education to examine the impact of the education of migrant workers on the health of their parents. We obtained similar results. However, endogeneity may still exist and thus make the estimation results biased and inconsistent. Therefore, we must consider the above results with caution. There are two main reasons for the potential endogeneity. On the one hand, there may be a problem of omitted variables. The level of education of individuals is often the result of decisions made by themselves and their parents, so the level of education of migrant workers may be related to unobservable ability and family background (17). These unobservable factors may also affect the health of parents, leading to the problem of omitted variables. For example, migrant workers with higher ability may not only obtain more education, but also master more health knowledge, and thus improve their own health and that of their families. If ability factors are omitted, the impact of the education of migrant workers on the health of their parents may be overestimated. On the other hand, there may be a problem of reverse causality. Health is a kind of capital that enables parents to obtain various resources. Without good health, not only is the parents' ability to obtain resources reduced, but other family members may also need to care for them, thereby affecting the opportunity and ability of these other family members to obtain resources and finally affecting the education of migrant workers (32).

To address these potential endogeneity problems in the benchmark regression, we first carefully selected control variables to maximize control of the relevant influencing factors. On this basis, we also adopted control variables for regional characteristics and community characteristics to reduce the problem of omitted variables caused by unobservable regional differences and community resource endowments. Despite these efforts, endogeneity problems may still exist. Therefore, instrumental variables for the education of migrant workers are also used to reduce bias and inconsistency that may arise due to endogeneity.

According to theory, effective instrumental variables must meet two conditions: first, they must be related to the education of migrant workers and second, they must not be directly related to the health of the parents of migrant workers. In this paper, the average length of schooling in the community, excluding the migrant workers' own years of education, is considered an effective instrumental variable for the education of migrant workers. On the one hand, the average length of schooling in a community to some extent reflects the educational resources and quality of the community, which affect the length of schooling of migrant workers (33). Therefore, there may be a positive correlation between the two. The results are shown in Table 4.

**Table 4:** Instrumental variable test (1): correlation test results.

**Explanatory variables**	**(1)**	**(2)**	**(3)**	**(4)**
	**Dependent variable: migrant workers'**			
	**years of education**			
The average length of schooling	0.996***	0.674***	0.835***	0.457***
in the community	(0.034)	(0.062)	(0.036)	(0.067)
Control variables	No	Yes	No	Yes
Province FE	No	No	Yes	Yes
Observations	13,552	3,686	13,552	3,686
F statistical value	861.918	46.686	57.935	20.789
Adj-R^2^	0.067	0.124	0.096	0.152

Robust standard errors are in parentheses, ***P < 0.01, **P < 0.05, *P < 0.1, respectively. The control variables (as shown in Table 2) and the constant terms are not listed.

The table shows that the average length of schooling in the community has a significant positive correlation with the education of migrant workers, regardless of whether the influence of fixed effects and other variables is controlled. That is, instrumental variables are significantly correlated with endogenous variables, so the correlation hypothesis of instrumental variables is supported. In addition, regardless of whether other variables and regional fixed effects are controlled, the F statistical values are all ≥10, indicating that there is no problem of weak instrumental variables (34).

On the other hand, the average length of schooling in the community (excluding that of migrant workers) cannot address unobservable family traditions, preferences, abilities, or other variables. It has a strong exogenous nature and is not related to the health of the parents of migrant workers at the micro level. In addition, considering the average years of schooling in the community significantly reduces the potential for measurement errors. The design of this instrumental variable has been adopted by others (35).

To test the exogenous nature of the instrumental variable, since only one instrumental variable is considered in this paper, the method proposed by Baron and Kenny (36) is adopted for testing according to the following steps: (1) The impact of average years of education in the community on the health of parents of migrant workers is tested. A significant coefficient indicates that the average length of education in the community has a significant impact on the health of parents of migrant workers. (2) The influence of the average years of education in the community on the education of migrant workers as an endogenous variable is tested. A significant coefficient indicates that the average length of education in the community is related to the education of migrant workers as an endogenous variable. (3) Based on step (1), the variable for the education of migrant workers is added. If the influence of the endogenous variable is significant and the coefficient of average years of education in the community is not significant compared to the coefficient in Step (1), the average years of education in the community as an instrumental variable of the education of migrant workers can only indirectly affect the health of parents through the education of the education of migrant workers' education. That is, it meets the exogeneity requirements. Since step (2) has been examined in Table 4 and the results are as expected, step (1) and step (3) are mainly investigated here. The results are shown in Table 5.

**Table 5:** Instrumental variable test (2): exogeneity test results.

**Explanatory variables**	**(1)**	**(2)**	**(3)**	**(4)**	**(5)**
	**SAH (1)**	**SAH (2)**	**SAM**	**Minus-7**	**Depressed**
**Part A**					
The average length of schooling in the community	0.038	0.082**	0.022	0.023	0.102*
	(0.038)	(0.038)	(0.042)	(0.035)	(0.055)
Control variables	Yes	Yes	Yes	Yes	Yes
Province FE	Yes	Yes	Yes	Yes	Yes
Observations	3,415	3,631	3,532	3,681	3,516
Wald statistical value	166.01	179.38	160.94	506.45	60.85
Pseudo-R^2^	0.048	0.049	0.057	0.117	0.041
**Part B**					
Migrant workers' years of education	0.016*	0.017*	0.025***	0.023***	0.027**
	(0.009)	(0.009)	(0.010)	(0.008)	(0.013)
The average length of schooling in the community	0.032	0.074*	0.009	0.012	0.090
	(0.038)	(0.038)	(0.042)	(0.035)	(0.055)
Control variables	Yes	Yes	Yes	Yes	Yes
Province FE	Yes	Yes	Yes	Yes	Yes
Observations	3,415	3,631	3,532	3,681	3,516
Wald statistical value	168.43	182.53	168.19	511.48	66.67
Pseudo-R^2^	0.049	0.050	0.059	0.118	0.044

Robust standard errors are in parentheses, ***P < 0.01, **P < 0.05, *P < 0.1, respectively.

Part A of Table 5 shows that when the education of migrant workers is not controlled, the average length of education in the community (excluding the migrant workers' own education) has a significant impact only on parents' SAH (2) and depression and not on parents' SAH (1), SAM, or calculation precision (Minus7). After the education of migrant workers is controlled, as shown in Part B, the average length of education in the community has a significant impact only on parents' SAH (2) and does not affect other health indicators. Therefore, in general, the average length of education in the community is exogenous to the health of parents. It should be noted that the relationship between the education of migrant workers and their parents' SAH (2) is temporarily not considered in the 2SLS regression analysis because, relative to SAH (2), the instrumental variables do not meet the exogeneity requirements.

Regarding the effect of migrant workers' education on the health of their parents, possible endogeneity has not been discussed in depth. However, according to our analysis, there may be an endogeneity problem, which would lead to inaccurate results. For this reason, the appropriate control variables were first carefully selected to reduce the influence of endogeneity as much as possible. Furthermore, 2SLS was used to address the endogeneity problem in a more comprehensively. The rationality of the instrumental variables was tested in detail and the instrumental variables selected in this paper were found to be appropriate. In this section, IV-probit regression is performed using the instrumental variable selected above. The results are shown in Table 6.

**Table 6:** 2SLS results.

**Explanatory variables**	**(1)**	**(2)**	**(3)**	**(4)**	**(5)**
	**SAH (1)**	**SAH (2)**	**SAM**	**Minus-7**	**Depressed**
Migrant workers' years of education	0.088	0.160**	0.044	0.051	0.195**
	(0.082)	(0.063)	(0.085)	(0.076)	(0.089)
Control variables	Yes	Yes	Yes	Yes	Yes
Province FE	Yes	Yes	Yes	Yes	Yes
Observations	3,415	3,631	3,532	3,681	3,516
Wald statistical value	182.00	231.05	163.24	498.46	81.34
Wald test	0.71	4.01**	0.05	0.13	2.48
*P*-test	0.399	0.045	0.825	0.723	0.115

Robust standard errors are in parentheses, ***P < 0.01, **P < 0.05, *P < 0.1, respectively. The control variables (as shown in Table 2) and the constant terms are not listed.

First, the endogeneity of migrant worker education is tested with the instrumental variables selected in this paper. The Wald test shows that, except for SAH (2), the null hypothesis was not rejected. That is, there was no endogeneity problem. This shows that the endogeneity problem has been well addressed by the control variables selected in this paper. Therefore, the results of the initial regression estimate are credible.

Although there is no endogeneity problem with respect to the education of migrant workers in this article, the IV-probit regression shows that an improvement in the education of migrant workers can significantly reduce the depression of their parents.

In summary, the estimation results of the baseline regression, robustness test and 2SLS method show that the education of migrant workers has a significant positive impact on the health of their parents, which verifies theoretical hypothesis 1.

## Further analysis

### Heterogeneity analysis

To better understand the relationship between the education of migrant workers and the health of their parents, this section further discusses whether the influence of the education of migrant workers on the health of their parents is heterogeneous in terms of parental education and age and whether they live with their children. The results are shown in Table 7.

**Table 7:** Heterogeneity analysis.

**Dependent variables**	**(1)**	**(2)**	**(3)**	**(4)**	**(5)**	**(6)**
	**Less than primary school**	**More than primary school**	**Under 60**	**Above 60**	**Cohabitation**	**No cohabitation**
SAH (1)	0.021**	0.012	0.018	0.016	−0.014	0.023**
	(0.010)	(0.017)	(0.012)	(0.013)	(0.022)	(0.010)
SAH (2)	0.028***	0.003	0.010	0.033**	0.045**	0.017*
	(0.010)	(0.017)	(0.012)	(0.013)	(0.023)	(0.010)
SAM	0.032***	0.010	0.020	0.031**	−0.002	0.029***
	(0.011)	(0.018)	(0.013)	(0.015)	(0.025)	(0.010)
Minus7	0.034***	−0.003	0.022**	0.032**	0.036*	0.021**
	(0.010)	(0.016)	(0.011)	(0.013)	(0.021)	(0.009)
Depressed	0.018	0.052**	0.033*	0.033*	0.034	0.030**
	(0.015)	(0.024)	(0.017)	(0.019)	(0.037)	(0.014)

Robust standard errors are in parentheses, ***P < 0.01, **P < 0.05, *P < 0.1. Control variables (as shown in Table 2), statistical indicators and constant terms are not listed.

To consider heterogeneity by educational level, because most parents have a primary school education, the sample is divided according to primary school education. Column (1) of Table 7 shows that the education of migrant workers has a significant impact on all health measures, except depression (depressed), for parents with a primary school or below education. Column (2) of Table 7 shows that the education of migrant workers has a significant impact only on the depression (depressed) of parents with an education higher than primary school. In general, there is significant heterogeneity in the health impact of the education of migrant workers according to the level of education of the parents.

For heterogeneity by age, since people enter old age at ~60 years of age, parents are divided into two groups with a cutoff at age 60. Column (3) of Table 7 shows that for the group of parents under 60 years of age, the education of migrant workers has a significant impact on two health indicators of parents, namely Minus7 and depression, but not on SAH (1), SAH (2) or SAM. Column (4) of Table 7 shows that for the group of parents 60 years of age or older, the education of migrant workers has a significant impact on four indicators of parents' health, namely SAH (2), SAM, Minus7, and depression, but without a significant impact on SAH (1). In general, the influence of children's education on their parents' health varies depending on the age of the parents.

In terms of whether migrant workers live with their parents, column (5) of Table 7 shows that for the group of migrant workers living with their parents, education has a significant impact on only two health indicators of parents, namely SAH (2) and Minus7, and it does not have a significant impact on SAH (1), SAM, or depression. Column (6) of Table 7 shows that for the group of migrant workers not living with their parents, education has a significant impact on all parents' health indicators. Therefore, the impact of the education of migrant workers on the health of their parents has significant heterogeneity. The results of the heterogeneity analysis support hypothesis 2.

### Influencing mechanism analysis

Currently, the way in which the education of migrant workers affects the health of their parents is unclear (17). The existing literature theoretically discusses the possible ways children's education affects the health of their parents and posits that children's education can improve the health of their parents by relaxing parental budget constraints and improving resource allocation and utilization efficiency. However, there are few empirical tests of this proposition.

Because budget restrictions are reflected primarily in an individual's economic situation and healthy productivity is achieved primarily by healthy behavior, this article will discuss the channels by which migrant workers' education affects their parents' health from the perspective of the economic situation of migrant workers and the healthy behavior of their parents. In view of the available data, this paper uses the family income (*IM*) and work type (*WK*) of migrant workers as indicators to measure their economic status and uses the length of sleep (*NST*), lunch break (*NSM*), strong exercise (*VS*), moderate exercise (*MS*), light exercise (*LS*), smoking (*SK*), alcohol consumption (*DK*) and physical examinations (*PE*) of their parents as indicators of healthy behaviors. According to Li's classification (37), this article divides the types of work into five categories according to the prestige of the profession from low to high, where 1 denotes jobs in agriculture, forestry, animal husbandry, fisheries and water conservancy production; 2 denotes commercial workers, personnel from the service industry and production and transportation workers; 3 denotes clerks; 4 denotes professional and technical personnel; and 5 denotes jobs in charge of a unit. As this paper studies migrant workers, the first type of occupation is excluded, while the others remain unchanged. The above information can be obtained from the CHARLS data in 2011 and 2013 and is not described here.

To effectively reveal the transmission mechanism, the intermediary effect test method proposed by Baron and Kenny (36) is adopted and a recursive model is established to test the mediating effect of economic status and the health behaviors of their parents' health behaviors. (1) To test the influence of the education of migrant workers on their parents' health, if the coefficient of education of migrant workers is significant, the education of migrant workers has a significant impact on their parents' health. (2) To test the impact of the education of migrant workers on their economic status and their parents' health behaviors as intermediary variables, if the coefficient of education of migrant workers is significantly positive, education is conducive to improving the economic status of migrant workers and their parents' health behaviors. (3) Based on the first step, we add the variables for the economic status and their parents' health behaviors. If the influence of the intermediary variables is positive and the coefficient of education of migrant workers is lower than in the first step or becomes insignificant, the economic status of migrant workers and the health behaviors of their parents have partial or full intermediary effects.

Based on the above test ideas, we set the following empirical model:

The first step is to test whether the education of migrant workers affects the health of their parents.


(2) $$\begin{array}{l} Pr\left(Y_{i}=i\right)=\phi \left(\alpha +\beta E_{i}+\gamma X_{i}+\mu_{k}+u_{i}\right) \end{array}$$

The second step is to test whether the education of migrant workers affects their economic status and the health behaviors of their parents.


(3) $$\begin{array}{l} Pr\left(IB_{i}=1\right)=\phi \left(\alpha +\beta E_{i}+\gamma X_{i}+\mu_{k}+u_{i}\right) \end{array}$$

The third step is to include variables of education, economic status, and parents' health behavior into the model at the same time.


(4) $$\begin{array}{l} Pr\left(Y_{i}=i\right)=\phi \left(\alpha +\delta IB_{i}+\beta E_{i}+\gamma X_{i}+\mu_{k}+u_{i}\right) \end{array}$$

The first model is the same as model (1), and the results shown in Table 2 are basically in line with the theoretical expectation. Therefore, these results are not described here in detail. *IB*_*i*_ represents the mediating variables: the economic status of migrant workers and the health behaviors of their parents [when the mediating variables are continuous variables, model (3) is carried out using the OLS method]. This part focuses on the second and third steps, and the results are shown in Tables 8, 9, respectively (all coefficients are marginal effects).

**Table 8:** Mechanism analysis: second step.

**Explanatory variables**	**(1)**	**(2)**	**(3)**	**(4)**	**(5)**	**(6)**	**(7)**	**(8)**	**(9)**	**(10)**
	**NST**	**NSM**	**VS**	**MS**	**LS**	**SK**	**DK**	**PE**	**IM**	**WK**
Migrant workers' years of education	0.024**	0.490*	−0.004	0.000	0.004	−0.005***	0.006***	0.004*	0.045***	0.100***
	(0.011)	(0.255)	(0.004)	(0.004)	(0.004)	(0.002)	(0.002)	(0.003)	(0.011)	(0.006)
Control variables	Yes	Yes	Yes	Yes	Yes	Yes	Yes	Yes	Yes	Yes
Province FE	Yes	Yes	Yes	Yes	Yes	Yes	Yes	Yes	Yes	Yes
Observations	4,259	4,314	1,724	1,722	1,711	3,300	4,487	4,412	3,414	3,594
Wald/F statistical value	4.935	16.765	212.68	180.35	61.76	892.22	856.86	260.34	10.850	10.782
Pseudo/Adj-R^2^	0.034	0.062	0.095	0.079	0.038	0.511	0.170	0.051	0.095	0.110

Robust standard errors are in parentheses, ***P < 0.01, **P < 0.05, *P < 0.1. The control variables (as shown in Table 2) and the constant terms are not listed.

**Table 9:** Mechanism analysis: third step.

**Explanatory variables**	**(1)**	**(2)**	**(3)**	**(4)**	**(5)**
	**SAH (1)**	**SAH (2)**	**SAM**	**Minus7**	**Depressed**
Migrant workers' years of education	0.004	0.000	0.003	0.002	−0.003**
	(0.004)	(0.004)	(0.003)	(0.004)	(0.002)
IM	0.025***	0.025***	0.024***	0.015*	0.007**
	(0.008)	(0.007)	(0.006)	(0.008)	(0.003)
WK	−0.005	−0.012	0.001	0.002	0.013**
	(0.013)	(0.012)	(0.010)	(0.013)	(0.005)
NST	0.022***	0.025***	0.015***	0.011**	0.015***
	(0.005)	(0.005)	(0.004)	(0.005)	(0.003)
NSM	0.000	0.000	−0.000	−0.000	0.000**
	(0.000)	(0.000)	(0.000)	(0.000)	(0.000)
SK	−0.056*	−0.031	−0.021	−0.017	0.001
	(0.033)	(0.029)	(0.025)	(0.033)	(0.015)
DK	0.059**	0.076***	0.000	0.025	−0.011
	(0.024)	(0.022)	(0.019)	(0.024)	(0.012)
PE	−0.031	−0.044**	−0.000	0.061***	0.010
	(0.023)	(0.020)	(0.017)	(0.022)	(0.011)
Control variables	Yes	Yes	Yes	Yes	Yes
Province FE	Yes	Yes	Yes	Yes	Yes
Observations	1,667	1,932	1,919	1,933	1,781
Wald statistical value	161.47	163.05	104.95	273.45	99.96
Pseudo-R^2^	0.088	0.089	0.069	0.128	0.122

Robust standard errors are in parentheses, ***P < 0.01, **P < 0.05, *P < 0.1. The control variables (as shown in Table 2) and the constant terms are not listed.

As shown in Table 8, the results of the second step test show that the education of migrant workers has a significant impact on the behavior of their parents in terms of health and their own economic situation. Migrant workers' education significantly improves their parents' sleep (night sleep and naps), alcohol consumption and physical examination behaviors, and their own economic status (income and work). However, the education of migrant workers also significantly reduced the possibility that parents do not smoke, which shows that the education of migrant workers may increase the probability that their parents smoke (38–41). However, the measurement of parents' smoking behavior is based on whether parents have smoked, not whether they currently smoke, so the indicator is not rigorous and may not accurately reflect the impact of the education of migrant workers. Therefore, the significance of this index is relatively limited. The coefficients for parental health behaviors, captured by hours of sleep at night and during lunch break, smoking, drinking, and physical examinations, and the economic status of migrant workers, captured by their income and work status, are significant. Therefore, in the third step we add these seven variables to analyze the mediation effect.

According to Table 9, the third step of the test shows that from the perspective of the economic situation of migrant workers, income and work type have a significant impact on the health of parents. From the perspective of parental health behaviors, the length of sleep at night, the length of lunch breaks, alcohol use, and physical examinations had significant effects on parents' health. Having an extended lunch break reduced parental depression (depressed), but this effect was significant only statistically and not economically (42–44). There was heterogeneity in the influence of physical examinations on the health of the parents. This may be because as parents age, physical examinations allow them to detect their own diseases, leading to an increased psychological burden and a lower subjective assessment of their own health. However, according to the objective health indicator of Minus7, the health of parents improved with participation in physical examinations, indicating that physical examinations can indeed improve the health of parents. Therefore, it is necessary to correctly recognize health changes to maintain physical and mental health.

In addition, parents who had never smoked before had a lower SAH (1). This may be because this indicator reflects the impact of family history on health. Long-term smoking behavior requires financial support and cannot be maintained with low family income, and family history is also an important factor affecting parents' health. As a result, parents' SAH (1) may be poor if they have never smoked. Therefore, we must be cautious when making inferences about these indicators of health behavior.

Additionally, after controlling the intermediary variables, compared to the coefficient estimation results regarding the education of migrant workers in model (2) (see Table 2), the impacts of the education of migrant workers in model (4) on health indicators, including parents' SAH (1), SAH (2), SAM, and calculation precision (Minus7), are no longer significant (see Table 9). Furthermore, as shown in column (5), the coefficient of the education of migrant workers on parents' depression (depressed) becomes negative. This shows that, on the one hand, the mediating variables selected in this article can explain why the education of migrant workers has a positive effect on the health of their parents (45–48). However, education of migrant workers can lead to an increase in parental depression. This may be because after controlling the economic and cognitive impacts of education on health, having a higher education may encourage migrant workers to move to distant areas where they may earn higher income but be separated from their parents for a long time, leading to increased parental depression.

In summary, children's economic ability, captured by their income and work type, and parents' health behavior, captured by their sleep (nighttime sleep and lunch break), alcohol consumption, and physical examinations, are important intermediary channels by which migrant workers' education affects their parents' health. This shows that migrant workers' education has a positive impact on their parents' health, mainly through relaxation of budget constraints and improving the efficiency of their parents' health production. The above results verify the hypothesis in the theoretical section.

## Conclusion and implications

Under the background of deepening population aging, rural areas are facing more severe pension problems, and the transfer of rural labor force to non-agricultural industries and cities makes this problem more prominent. At the same time, because the non-agricultural employment labor market is more fully developed and the degree of marketization is higher, human capital has become a key factor affecting individual employment performance. To this end, this paper uses 2011 and 2013 China Health and Retirement Longitudinal Study (CHARLS) data to try to examine the impact of migrant workers' education on parents' health from a family perspective. The following results are obtained. Migrant workers' education has a significant positive impact on their parents' health. Heterogeneity analysis shows that there are significant differences in the impact of migrant workers' education on the health of parents from different groups. This positive effect has a greater impact on the health of parents who are older, less educated, and do not live with their children. Mediation analysis shows that children's economic ability, captured by income and type of work, and parents' health behavior, captured by sleep, alcohol consumption, and physical examinations, mediate the relationship. Therefore, the education of migrant workers affects the health of their parents mainly by relaxing budget constraints and improving the efficiency of the health production of their parents. In addition, this paper also found that when the mediating effect of children's economic resources and parents' health behavior is controlled, the education of migrant workers may significantly increase parents' depression, but it does not affect other health indicators.

The results of this study show that children's education is universal in improving parents' health. Previous studies have found that children's education can improve parental health for the general population (9, 17). However, there is no research on migrant farmers. For this group, in order to obtain economic income, they have to leave the family, resulting in the inability to give their parents daily care and emotional comfort, and can only rely on economic resources to meet their parents' health needs (4). However, the results of this study show that the improvement of children's education on parents' health is not limited by time and space. Even if they cannot physically live together, children's education can still improve parents' health. This is consistent with the findings of Liu (21). They believe that the damage to parents' welfare is not caused by the departure of children, but by the disappearance of social networks among family members. As long as there is a social network between family members, even if the family members are not together, children can still improve parental welfare.

Different from previous studies, this paper further examines the initiative of parents in the process of children exerting influence on parents. The results show that the improvement of children's education on parents' health will be affected by parents' initiative. Those parents who are young, highly educated, and live with their children have higher ability to improve their own health, and they do not need too much support from their children (4), which makes their children's education have less impact on the health of such parents. However, those parents who are older, less educated and unable to live with their children are limited by their own abilities, and they need their children's support even more (9), which makes children's education have a greater impact on the health of such parents.

In the process of analyzing the impact mechanism, the research in this paper found that economic resources play an important role in the process of migrant farmers' education affecting their parents' health, which is consistent with the research conclusion of Ma (17). Additionally, further analysis in this paper found that in addition to economic resources, improving parental health behavior also played an important mechanism role. When the intermediary effect of children's economic resources and parents' health behaviors is controlled at the same time, the influence of migrant workers' education on many indicators of parents' health basically disappears, which indicates that migrant workers' education mainly achieves the purpose of improving parents' health through their economic resources and parents' health behaviors. In addition, this paper also found that when the intermediary effect of children's economic resources and parents' health behavior is controlled at the same time, the education of migrant workers will lead to a significant increase in parents' depression, which indicates that although education can bring positive effects such as the improvement of economic ability, it may also have negative effects. However, previous studies on general groups focused mainly on the former and neglected the latter. The research on migrant farmers in this article provides a natural advantage in identifying the negative effects of education. The reason is that education can improve the economic income of migrant farmers, mainly by forcing them to leave their hometown and go to cities to look for better employment opportunities. However, this will lead to the separation of parents and children, which will easily cause parents to miss their children, and then induce parents' depression.

Although some new discoveries have been made, there are still some shortcomings in this paper that need to be improved. First of all, this paper only uses the years of education to measure education, but fails to discuss the influence of different majors and different quality of education. Second, research on the influence mechanism is limited to the family, and the influence of social capital, such as neighborhood interaction, is not discussed. According to the availability of data, the author will continue to focus on this issue.

Based on the above analysis, this paper highlights the importance of developing education in rural and poor areas. Then, as an externality of education, the health of individuals and their family members can be improved, which in turn can promote the transfer of rural labor to non-agricultural industries and cities, improve the supply of labor, curb the rapid rise of labor costs, and promote healthy economic development.

## Data availability statement

The original contributions presented in the study are included in the article/supplementary material, further inquiries can be directed to the corresponding author.

## Author contributions

KY: writing—original draft. SF: conceptualization, supervision, writing—review and editing, data curation, visualization, and investigation. Both authors contributed to the article and approved the submitted version.

## Funding

This study is supported by the National Social Science Fund of China in Education (Western Project): Research on the Mechanism and Countermeasures of Financial Investment in Compulsory Education to Promote the Equal Development of Human Capital of Rural Adolescents in Western China (Grant No. BFX220336).

## Conflict of interest

The authors declare that the research was conducted in the absence of any commercial or financial relationships that could be construed as a potential conflict of interest.

## Publisher's note

All claims expressed in this article are solely those of the authors and do not necessarily represent those of their affiliated organizations, or those of the publisher, the editors and the reviewers. Any product that may be evaluated in this article, or claim that may be made by its manufacturer, is not guaranteed or endorsed by the publisher.
